# A novel microfluidic device with parallel channels for sperm separation using spermatozoa intrinsic behaviors

**DOI:** 10.1038/s41598-023-28315-7

**Published:** 2023-01-21

**Authors:** Ali Heydari, Mohammad Zabetian Targhi, Iman Halvaei, Reza Nosrati

**Affiliations:** 1 Faculty of Mechanical Engineering, Tarbiat Modares University, Tehran, Iran; 2 Faculty of Medical Sciences, Tarbiat Modares University, Tehran, Iran; 3 Department of Mechanical and Aerospace Engineering, Monash University, Melbourne, Australia

**Keywords:** Biomedical engineering, Mechanical engineering, Cellular motility

## Abstract

Isolating high-quality motile sperm cells is considered to be the main prerequisite for a successful artificial pregnancy. Microfluidics has emerged as a promising platform capable of mimicking in-vivo environments to separate motile sperm cells and bypassing the need for the current invasive clinical sperm separation methods. In this study, the proposed microfluidic device exploits the parallelization concept through symmetry to increase both the processed sample volume and the injected flow rate compared with the previous conventional devices, which used rheotaxis as their primary method of sperm separation. Using the finite element method (FEM) and flow simulations, the trajectories of sperm cells exhibiting rheotaxis behavior were predicted inside the proposed device. Different flow rates, including 0, 0.5, 1.5, 3, 4.5 and 6 μl/min, were experimentally injected into the device, and the effect of flow rate on the size of the hypothetical rheotaxis zone and the number of isolated sperm cells was investigated. Furthermore, it was illustrated that 100% of the isolated motile sperm cells are motile, and by manipulating the injected flow rate into the device, different classes of sperm cells in terms of motility parameters can be separated and utilized for further uses.

## Introduction

Sperm motility is required for sperm cells to swim through the female reproductive tract to reach the ovum and fertilize it^1,2^. Based on statistical data provided by WHO, 10–15% of couples worldwide (more than 50 million) experience failure in conceiving naturally^3,4^, and almost 50% of these infertility cases are attributed to male infertility due to the low sperm motility and deficient count^5^. To overcome such setbacks, assisted reproductive technologies (ARTs) such as intrauterine insemination (IUI), in vitro fertilization (IVF), and intracytoplasmic sperm injection (ICSI) are commonly used to achieve pregnancy. However, the success rate for the mentioned methods has remained fixed merely at 30–40% per cycle^6^, indicating the need for novel and more effective tools and techniques for increasing the chance of pregnancy while preventing possible risks.

Prior to mentioned clinical resolutions, high-quality and motile sperm cells must be selected^7,8^. Currently, two main methods exist for separating sperm with the desirable features mentioned above, such as swim-up and gradient centrifugation. Both methods require the semen sample to be centrifuged at high rotating velocities, which is detrimental to sperm's morphology, and DNA integrity^9–11^; Centrifugation also enters reactive oxygen species (ROS) into the sample, which threatens the viability of sperm cells even more^12–14^.

Microfluidic systems are promising platforms for fluid manipulation at small scales (1 um–1 mm), which could be exploited to replace the traditional methods for successfully separating and analyzing motile sperm^15,16^. In contrast to these methods, microfluidic systems have entirely bypassed the need for semen sample centrifugation without invading sperm’s natural barriers and damaging their precious cargo (i.e., DNA)^17–19^. These systems have other advantages, including low sample consumption and removing human error due to automation capability^20,21^.

Different microfluidic devices have been designed and experimented on to replace the initial process of healthy sperm separation prior to ARTs (swim-up and centrifugation methods)^22–25^. These devices can be categorized into two groups generally. The first group is active microfluidic devices that use external forces like optical traps, electrophoresis, and hydrostatic pressure^24,26,27^. Even though these techniques may provide a significant dominance on the sample, they are not only unsuitable for the isolation of healthy sperm due to their invasive mechanism but also include complicated experimental setups, making them expensive and time-consuming^14,20^. In the second category, the passive microfluidic systems, the separation process is based on sperm's intrinsic behavior like boundary-following and response to external stimulants such as fluid flow, temperature, and chemical gradients^28–32^. The passive systems can separate sperm without damaging its morphology or DNA integrity by mimicking the in vivo environment, allowing for the best and most competent sperm to be selected^14^.

Different studies have focused on these intrinsic behaviors. In one study, a microfluidic device was developed that investigated the tendency of sperm to swim near walls^31^, and its concept was then used by Nosrati et al.^22^ and Simchi et al.^33^ to develop a high-throughput device for sperm separation. Another experimental study provided insight into the rheotactic behavior of sperm (i.e., the ability to reorient and swim against the fluid flow direction)^34^; its concept was then used in microfluidic devices to separate motile and progressive sperm from the initial sample^35,36^. Other studies have focused on different abilities and features of sperm cells, culminating in the fabrication of different devices for sperm separation based on thermotaxis and chemotaxis (kinematic response to temperature and chemical gradients, respectively)^29,30^.

Although the devices that work based on sperm cells’ intrinsic behavior may ensure the intactness of sperm DNA, the flow rate injected into them must be relatively small to allow the sperm cells to exhibit their innate features. This comes with the penalty of decreased sample volume processed compared with high-throughput devices, such as spiral microfluidic chips, which are capable of handling large sample volumes^37^. However, it should not be forgotten that spiral devices may impose the risk of DNA damage to sperm cells due to the high shear rate they exert on particles, which may reduce the chances of artificial pregnancy when the separated sperm are used for infertility treatment^6^. Moreover, spiral microchips separate sperm from round cells, such as RBCs and WBCs, merely based on their physical properties (i.e. density and diameter)^38^. Also, the biological entities accumulated inside their outlets are not 100% pure. In comparison, the sperm cells separated by employing intrinsic features may have higher quality in terms of morphology, motility, DNA integrity, viability, vitality, and of course, 100% purity in the designed device’s outlets.

Therefore, this study proposes a device that simultaneously exploits two main features of a healthy and progressively motile sperm cell, rheotaxis and boundary-following behavior, to separate sperm cells from debris or dead cells passively. Also, by employing the parallelization concept, the volume of the processed sample and the injected flow rate were increased two times the existing devices that use rheotaxis as their primary technique for separation. Previous devices mainly used one microchannel for sperm separation; therefore, the sample injection flow rate was relatively low. This device aimed to exploit the advantage of using arrays and parallel microchannels to overcome this shortcoming. Additionally, finite element method (FEM) simulations were used to model the flow inside our device, obtain the velocity field and flow shear rate in the rheotaxis zone, and simulate the immotile particles inside our device. A MATLAB^39^ code was also developed that couples the velocity field and shear rate obtained by the COMSOL^40^ to predict the reorientation of sperm against the flow direction by solving the sperm motion equations. Moreover, a range of flow rates was experimentally injected into the proposed device to observe the rheotaxis phenomenon and to assess the proposed device's ability to separate different classes of human sperm in terms of motility and swimming velocity. Finally, the results show that the separated sperm through rheotaxis were 100% motile and capable of exploiting boundary-following behavior to reach the target reservoirs to be extracted from the device for further use.

## Result and discussion

### Mechanism of sperm reorientation (rheotaxis) and simulations results

Sperm must swim within the female reproductive system over a long distance, compared with their length, to fertilize an oocyte^41^. A sperm moves in a roughly circular pattern in the absence of external fluid flow^42^. Nonetheless, when an external flow is introduced, the sperm is reoriented upstream against the flow due to the fluid’s drag and torques exerted on both sperm’s head and tail. This mechanism, also known as rheotaxis, acts as a navigation system for sperm to locate and fertilize the oocytes^41^. Myriads of reports suggest that the rheotactic behavior occurs within a specific shear rate range or flow velocity. It has been shown that the shear rates between 2 to 5 s^−1^ and flow velocities between 22 to 102 μm.s^−1^ induce this behavior in human sperm^28,34,42^. In other words, shear rates/flow velocities lower than the minimum of the mentioned range seems not to affect sperm orientation. In contrast, values higher than the maximum of the mentioned range will only cause the sperm to be washed along the flow direction.

**Figure 1:**
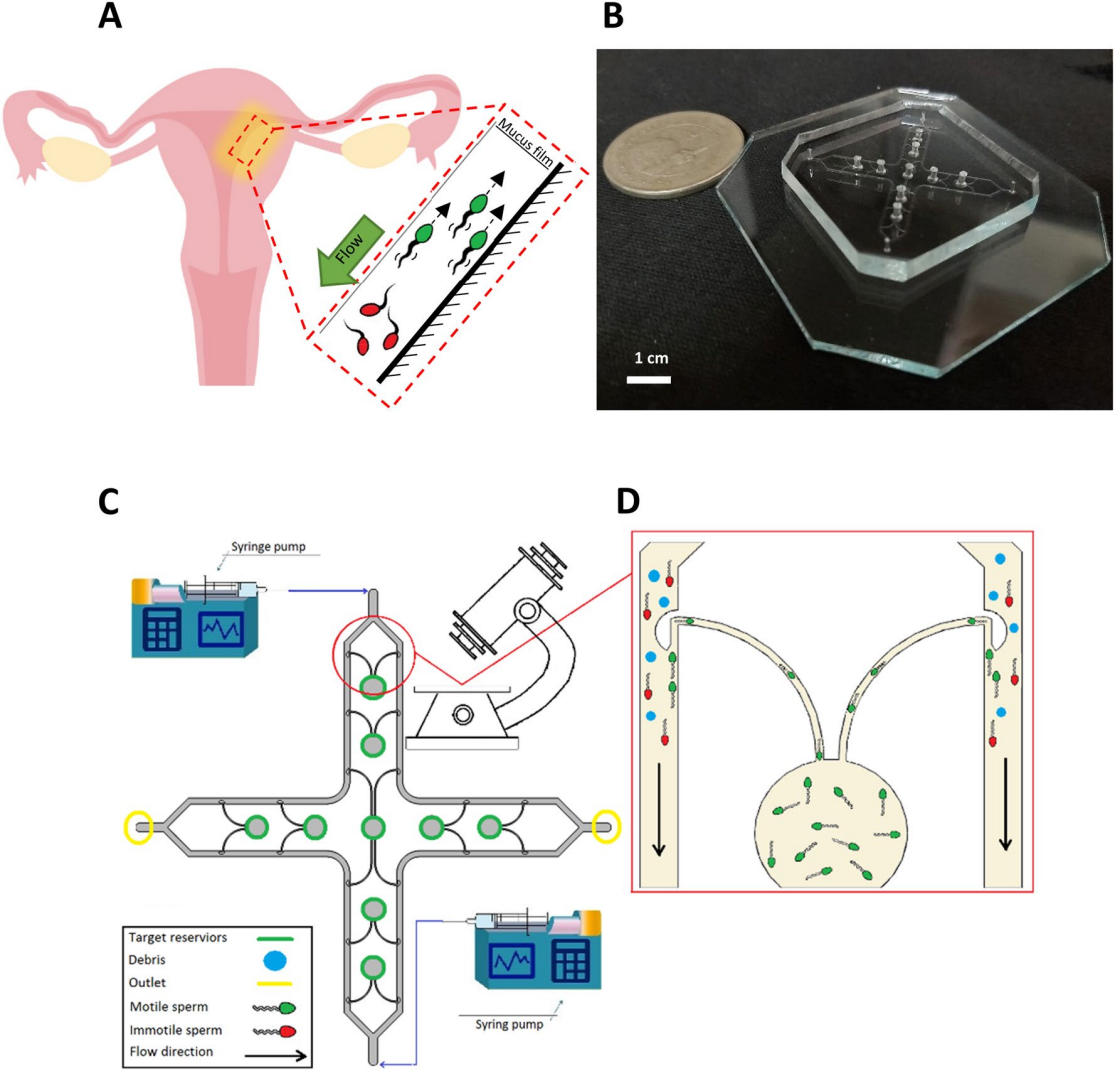
Microfluidic device for sperm separation. (**A**) Basics of sperm separation mechanisms inside the female reproductive tract. Mucus flow over the uterus walls will wash the immotile sperm cells away (shown in red). In contrast, motile sperm cells (shown in green) will employ rheotaxis and boundary-following behavior to swim against the flow direction and move toward the oocyte. (**B**) The microfluidic device. (**C**) Mechanism of sperm separation in the proposed microchip inspired from the uterus walls. The obstacles placed along the walls create a rheotaxis zone in their frontal area and facilitate the entrance of healthy sperm into the narrow curved channels. Then by employing the boundary-following behavior, sperm cells are guided to reach the target reservoirs. (**D**) Schematics of the proposed device's performance. Part A was generated using Edrawmax^43^.

For exploiting the rheotactic behavior to separate motile sperm and to increase the input flow rate of the semen sample medium, a microfluidic device with four main parallel channels and nine target reservoirs is proposed. Inside the main channels, multiple hollow obstacles are placed along the walls to create a low-velocity area in front of them (rheotaxis zone), suitable for motile sperm cells to reorient themselves and swim against the flow direction to enter the hollow section while immotile cells (dead sperm, round cells, debris) are washed away. The hollow regions are connected to the target reservoirs through narrow curved channels. The role of these narrow channels is to guide the separated sperm (through rheotaxis) to reach the target reservoirs by employing boundary-following behavior. The width of main channels, the outer radius of hollow obstacles, the width of narrow channels, and the depth of microchannels are 500 μm, 300 μm, 90 μm, and 100 μm, respectively (Fig. 1) (also see Section I, SI).

As demonstrated in Fig. 1D, by employing the obstacles placed along the main channel walls, a rheotaxis zone can be created in the obstacle's frontal area. Motile sperm (shown in green) reorient against the flow direction and try to enter the narrow curved channels. If their swimming velocity prevails over the flow’s velocity, they can enter it to continue their path to reach the target reservoirs. However, dead or immotile sperm (shown in red) and other cells (shown in blue) are washed along the flow direction (also see Fig. S1 and Movie S1, SI). Therefore, to create a low-velocity zone in front of the obstacles, the injected flow rate of the semen sample must be calculated in a precise manner.

In addition to the FEM simulation, Adler's equation has been used to model the trajectories of reorienting sperm. When sperm is placed inside a viscous flow in a microchannel, its head will be closer to the walls and less affected by the flow. Due to the shape of the fluid flow’s velocity profile, a rotating torque is applied to sperm, causing it to rotate against the flow direction. Meanwhile, the propulsive force generated through flagellar beating helps the sperm move inside the flow. The angular velocity of the mentioned torque is calculated using Eq. (1), in which $\gamma$ is the shear rate of fluid flow near the wall, and $A$ is a constant related to the type of swimmer. Also, to take the effect of fluid flow over the reorienting sperm into account, the values of fluid flow velocity in X and Y directions have been extracted and related to swimming velocity (Eq. (2)). Lastly, Eq. (3) was used to track the reorienting sperm.

1 $$\omega =\frac{d\theta }{dt}=-A\gamma sin\theta$$

2 $${\overrightarrow{V}}_{swim}={\overrightarrow{V}}_{sperm}+{\overrightarrow{V}}_{fluid}, \left|{V}_{sperm}\right|=const.$$

3 $$\frac{{d\overrightarrow{r}}_{sperm}}{dt}={\overrightarrow{V}}_{swim}$$

**Figure 2:**
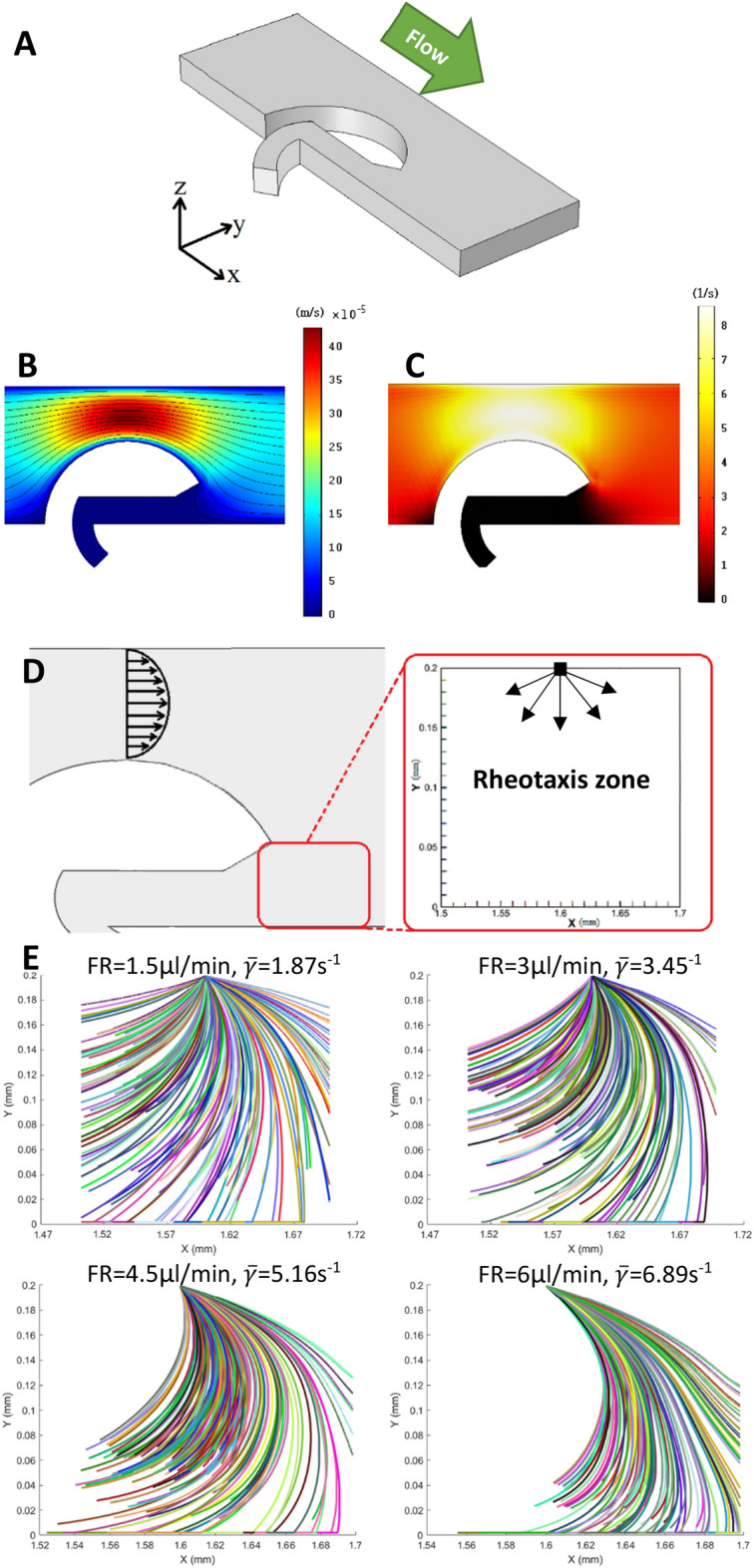
Simulation results of the velocity field, shear rate in the vicinity of the obstacles for flow rate 3 μl.min^−1^, and sperm trajectories inside the rheotaxis zone. (**A**) The geometry of a section of the main channel near one obstacle. (**B**) Velocity field of the sample medium over a cutplane with a 50 μm distance from the top surface with a maximum of 450 μm.s^−1^ and a minimum of zero. (**C**) The shear rate distribution over a cutplane with a 10 μm distance from the top surface with a maximum of 8.4 s^−1^. (**D**) A portion of the main microchannel and the hypothetical rheotaxis zone placed in front of the obstacle. The sperm are released into this zone from the top point shown. (**E**) predicted trajectories for motile sperm showing rheotactic behavior. The equations of sperm motion were solved for 150 sperm with a random initial velocity between 40–90 μm.s^−1^ and a random entrance angle into the rheotaxis zone between 0 and 180 degrees for injected flow rates starting from 1.5 to 6 μl/min. As the mean flow shear rate (flow velocity) inside the rheotaxis zone ($\overline{\gamma }$) increases, the number of sperm capable of resisting flow and reorienting against the flow direction decreases. Part B, and C are obtained using COMSOL^40^. Part E is obtained using MATLAB^39^.

To acquire a range of appropriate injected flow rates for the medium sample into the device and to obtain the velocity and shear rate distribution inside the rheotaxis zone, FEM simulations were carried out. Figure 2A–C depict a part of the main channel where an obstacle is placed, the velocity, and the shear rate distribution for a flow rate of 3 μl/min, respectively. In Fig. 2B, the velocity of the medium sample is extracted on an XY cut-plane with a distance of 50 μm from the top surface. The maximum velocity of the sample medium is calculated to be equal to 450 μm.s^−1^, represented in red color. In contrast, the minimum flow velocity is shown in the dark blue and is equal to zero. Also, considering flow streamlines passing over the obstacle, it can be inferred that the flow is stationary in the narrow curved channels, which facilitates the use of boundary-following behavior for the separated sperm through rheotaxis. Also, since the debris depend on flow to move within the channels, debris will not enter the narrow curved channels due to the absence of flow inside them. Figure 2C illustrates the shear rate distribution on an XY cut-plane with a distance of 10 μm from the top surface. As was mentioned in Eq. (1), the shear rate plays a prominent role in the swimming direction of sperm and how it varies. Also, since the sperm cells tend to swim adjacent to walls, the shear rate was extracted in the vicinity of the wall. The maximum shear rate is equal to 8.4 s^−1^, shown in yellow, and its minimum is equal to zero inside the narrow curved channels. In Fig. 2D, the hypothetical rheotaxis zone is designated in front of the obstacle and at the entrance of the narrow curved channel. As can be seen, the sperm cells are released from the point on the top center of this zone with a random velocity between 40 to 90 μm.s^−1^ and a random entering angle of 0–180º.

To predict the trajectories of sperm cells showing rheotactic behavior, the methodology developed by Zaferani et al.^20^ has been adopted with the difference that the lateral head movement, which was modeled using white Gaussian noise, is omitted since sperm cells are large swimmers compared with other swimming particles, and the role of random head fluctuations can be neglected^35^. Figure 2E shows the predicted sperm trajectories for four flow rates (shear rates) (also see Fig. S2). As shown in this figure, when the average shear rate exceeds the minimum threshold, the sperm cells can exhibit rheotactic behavior to change their swimming direction and swim against the flow. However, the role of flow velocity cannot be overlooked. As mentioned in Eq. (2), when the flow rate increases, the sperm cells must overcome a higher flow velocity to still move forward toward the entrance of the narrow curved channel. Thus, by increasing the injected flow rate, only the sperm cells with enough motility and swimming velocity that can resist the respective flow rate can enter the narrow curved channel to reach the target reservoirs. Another interesting point observed in the simulations, is that how the bottom wall can help sperm cells swim toward the narrow channel even at the highest injected flow rate (i.e. 6 μl/min). Since the flow velocity decreases near the bottom wall, the strongest sperm cells still have the opportunity to enter the narrow channels by simply continuing to swim in proximity to the bottom wall.

Concerning the limitations of the proposed model, the present framework for predicting sperm cells’ rheotactic behavior did not consider the effects of background flow physics on sperm cells' motion. The fresh semen sample contains shear-thinning non-Newtonian fluid, which is less viscous in the regions exposed to high viscous-based tensions^44^. This is especially important from two aspects; the first aspect is that the flow will be less viscous due to the viscous-gradient effects near the microchannel walls, where most sperm cells tend to swim. Also another important aspect is the oscillation of sperm cells' tails within the flow. This feature is effective since the wave propagation inside the spermatozoa tail will create a less viscous medium in its vicinity. This model, however, considered sperm cells as points. Therefore, implementing a shear-thinning non-Newtonian flow model into the FEM simulations and the proposed model will greatly enhance the accuracy of predicting sperm cells’ pathlines.

### Experimental results of sperm separation

**Figure 3:**
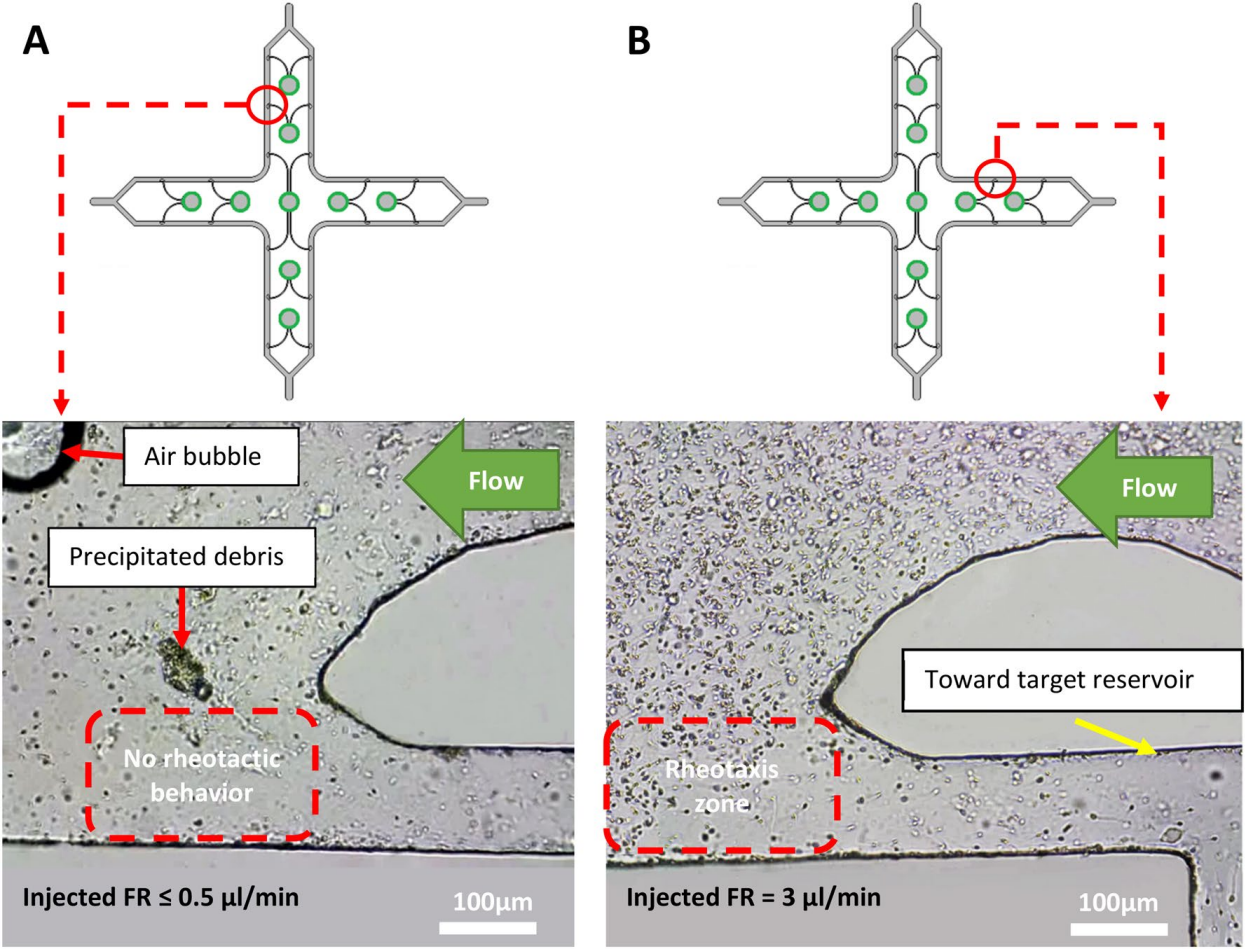
Rheotaxis-based separation of motile sperm. (**A**) When the injected flow rate was less than 0.5 μl/min, none of the motile sperm displayed rheotactic behavior either inside the main channels or the rheotaxis zone. (**B**) At flow rates larger than 0.5 μl/min, such as 1.5 and 3 μl/min, the motile sperm swam against the flow direction inside the rheotaxis zone and entered the narrow curved channels.

In order to investigate both the microchip's correct performance in separating motile sperm and the effect of flow rate/shear rate on the rheotaxis-based separation, the proposed device has been tested with different flow rates. Six flow rates were tested, including 0, 0.5, 1.5, 3, 4.5 and 6 μl/min. It should be noted that a syringe pump with dual slots was used to inject the sample into the device. Hence, two syringes with the above flow rates were used each time. However, it is possible to use only one syringe and double the mentioned flow rates, provided that a Y-type fitting is used to ensure an equal flow bifurcation to have an equal flow rate injected into each inlet. Based on the obtained results in flow rates 0 and 0.5 μl/min, none of the existing motile sperm swam in any specific direction, which can be attributed to the shear rate being lower than the minimum cut-off inside the rheotaxis zone in front of the hollow regions (Fig. 3A). Based on the simulations, the shear rate value inside the rheotaxis zone for flow rates 0 and 0.5 μl/min was equal to 0 and 1.34 s^−1^, respectively, which is lower than the minimum shear rate needed to encourage rheotactic behavior as reported by previous studies. However, when the injected flow rate was increased to 1.5 μl/min and higher, the motile sperm started to exhibit rheotactic behavior to enter the narrow curved channels and continue reaching target reservoirs (Fig. 3B) (also see Movie S2, SI).

**Figure 4:**
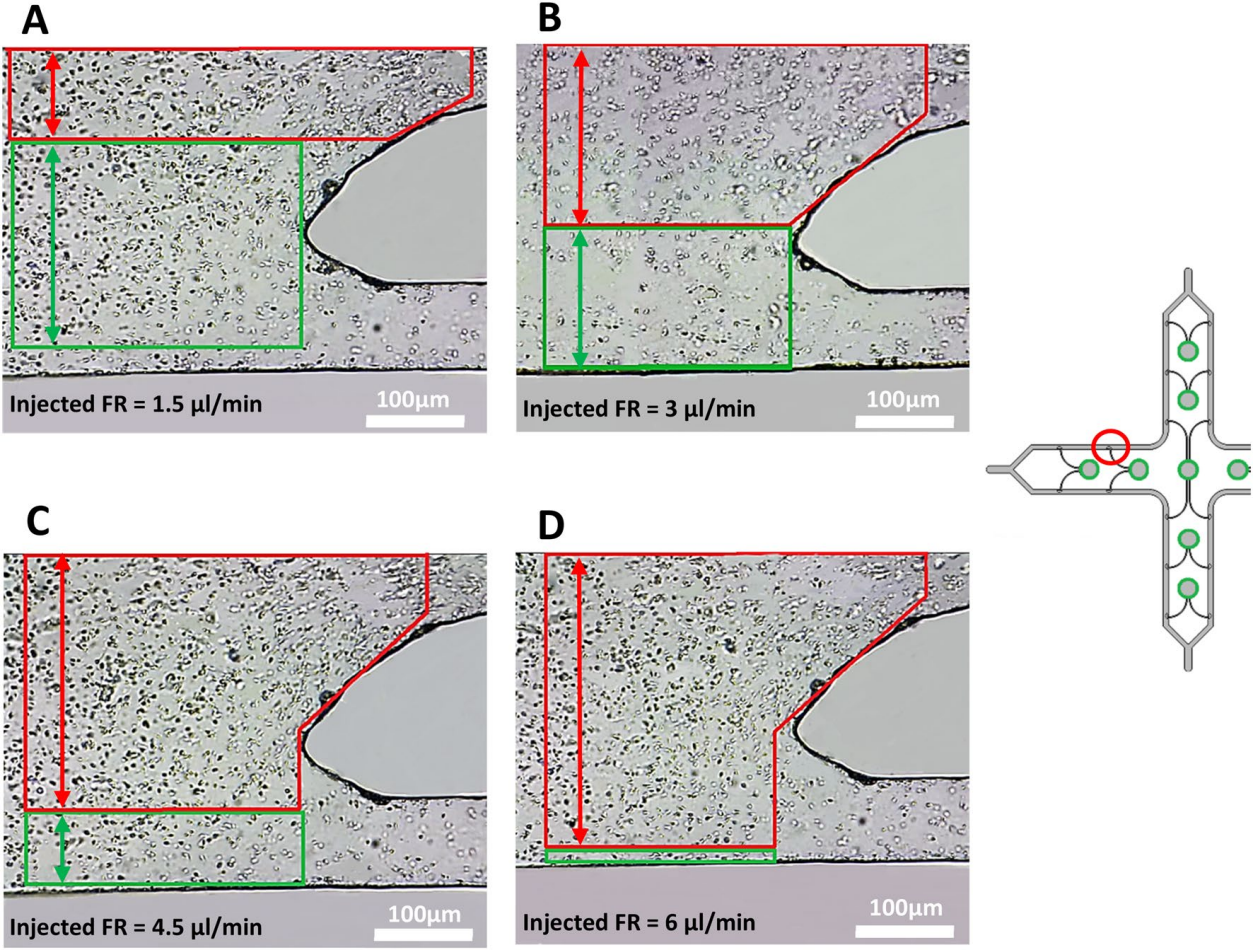
Inertia zone (red) vs. Rheotaxis zone (green). As the injected flow rate increases from 1.5 μl/min to 6 μl/min, the inertia zone prevails over the rheotaxis zone and causes it to eventually fade at 6 μl/min. This observation shows that the rheotaxis zone is not a fixed area inside the microchip and highly depends on the flow rate injected into the device.

By increasing the flow rate to 1.5 μl/min, the sperm started to reorient and swim against the flow direction. In this flow rate, a portion of the motile sperm entered the hollow region to continue their way to the target reservoirs (Fig. 4A). An interesting point was observed after increasing the injected flow rate from 1.5 to 3 μl/min and higher. In contrast to simulations where the rheotaxis zone was assumed to be a fixed region for all of the injected flow rates, it was observed that by increasing the flow rate, the inertia zone (shown in red) prevails over the rheotaxis zone (shown in green) and starts to expand as the size of the rheotaxis zone gradually decreases. At a flow rate of 3 μl/min (Fig. 4B), the rheotaxis zone is placed in front of the hollow region, making it possible to guide the maximum number of motile sperm into the hollow region and their respective target reservoirs precisely. Another interesting observation is that the rheotaxis zone almost fades away by increasing the injected flow rate to 6 μl/min. The number of motile sperm capable of swimming against a flow with such velocity drastically decreases (Fig. 4C,D).

**Figure 5:**
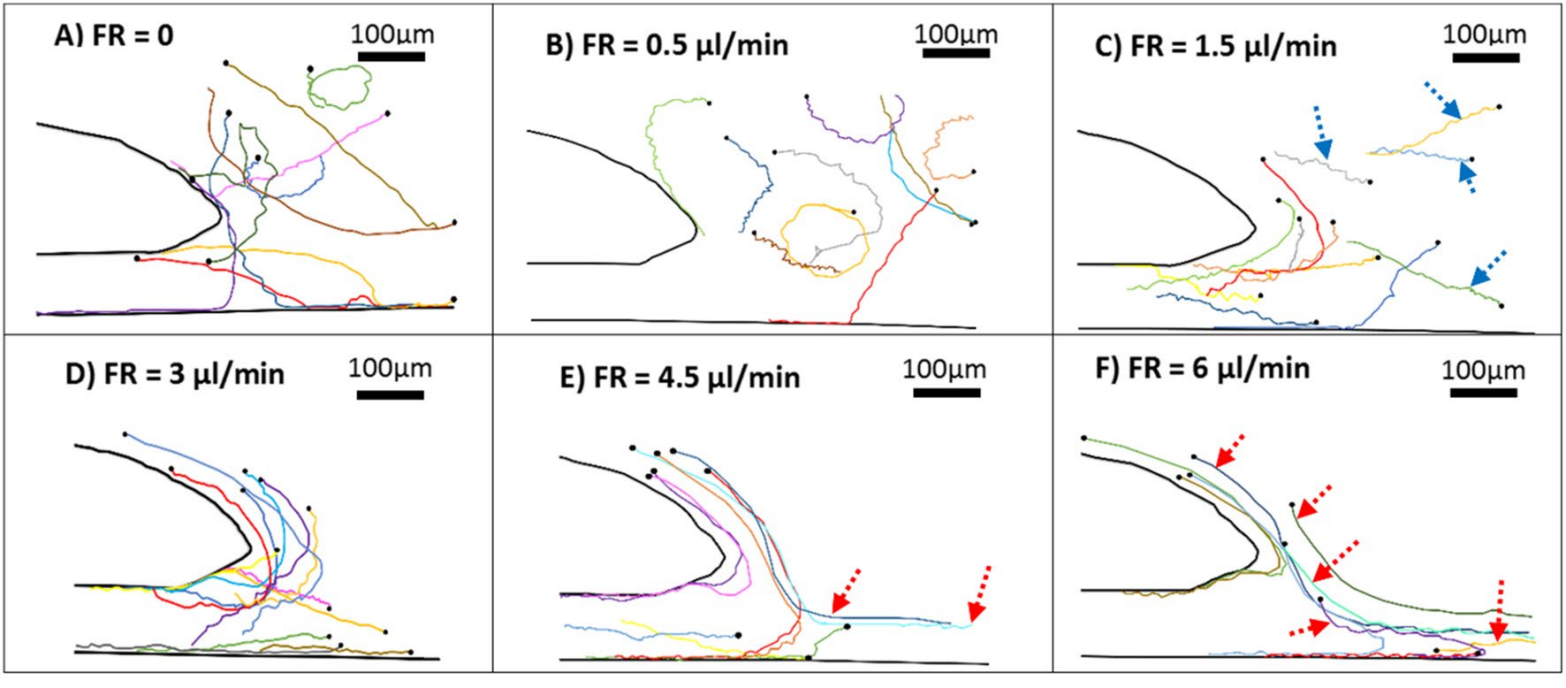
Motile sperm tracking history starting from the black circle. (**A**) Injected flow rate = 0, (**B**) Injected flow rate = 0.5 μl/min, (**C**) Injected flow rate = 1.5 μl/min; the blue arrows indicate the motile sperm which were able to resist the injected flow rate to swim inside the main channel rather than the rheotaxis zone, (**D**) Injected flow rate = 3 μl/min, (**E**) Injected flow rate = 4.5 μl/min, (**F**) Injected flow rate = 6 μl/min, The red arrows indicate the sperm cells reoriented against flow direction which were not able to resist the flow and were washed along the flow direction. Obtained using ImageJ^45^.

The trajectories of motile sperm were extracted to better visualize the effect of injected flow rate on the motion of motile sperm. As shown in Fig. 5A,B, for flow rates 0 and 0.5 μl/min, the motile sperm moved entirely in random or circular paths. As the flow rate was increased to 1.5 μl/min, motile sperm started to exhibit rheotactic behavior (Fig. 5C). However, a significant subpopulation of motile sperm (~ 40%) (shown with blue arrow) could swim against the flow in the main section of the channel rather than entering the hollow region, indicating that this flow rate is not large enough to push the motile sperm into the designated rheotaxis zone. By increasing the flow rate to 3 μl/min, the rheotaxis zone was pushed precisely in front of the hollow region. Almost 100% of the motile sperm that entered the rheotaxis zone could move directly toward the entrance of the narrow curved channels (Fig. 5D). Finally, as the injected flow rate was increased to 4.5 and 6 μl/min, the number of motile sperm capable of entering the narrow curved channels decreased again (Fig. 5E,F). An interesting point regarding these flow rates is that although a number of sperm cells displayed rheotactic behavior and managed to reorient themselves against the flow direction, the flow velocity was too high for them, and the mentioned sperm cells (shown with red arrow) could not overcome such flow and were washed along with the flow. Nevertheless, some of the sperm cells were still able to swim in the vicinity of the wall and exploit the lower flow velocity to reach the curved channel, which was also observed in the simulations.

**Figure 6:**
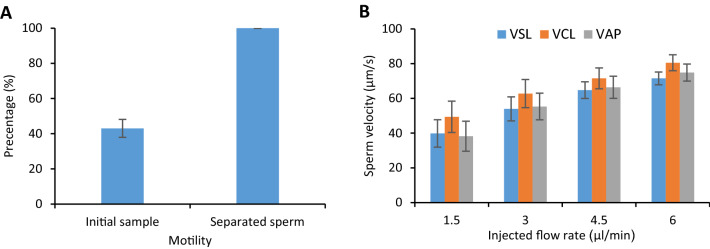
Separated human sperm quality analysis using CASA. (**A**) Separated sperm motility compared with the motility of the initial sample. (**B**) Velocity parameters of the separated sperm, including VAP, VCL, and VSL, for different flow rates. Values are reported as mean ± s.d (n ≥ 20, n: the number of single sperm cells analyzed.).

The size of the rheotaxis zone changes when the injected flow rate is increased or decreased. Not only does this affect the number of separated sperm, but it also has a determining role in the quality of the separated sperm. After testing the device with fresh human samples at each flow rate, the progressive motility parameters of the separated sperm were evaluated using computer-aided sperm analysis (CASA). Based on the obtained results, the motility of the separated sperm reached 100%, which shows roughly a 60% increase compared with the initial sample's average motility (Fig. 6A). Also, by examining parameters such as VSL (velocity along the straight line between the first and last point of the path), VAP (velocity along the average path), & VCL (velocity of sperm moving along the original path) (Fig. 6B), it can be concluded that the proposed device is capable of sorting the separated sperm cells based on their motility rate and swimming velocity. Therefore, it is enough to increase the injected flow rate to isolate spermatozoa with higher motilities from a sample so that only the sperm cells capable of overcoming the flow velocity at their respective flow rate can separate from the rest of the sample.

As depicted in Fig. 6A, 100% of the separated sperm were motile and showed a great tendency to enter the narrow curved channels and continue their way to the target reservoirs using boundary-following behavior (see Movies S3, S4, SI). The number of isolated sperm inside the target reservoir was counted to obtain the proposed device’s efficiency in terms of the number of isolated motile sperm. This device is capable of separating an average of 1.19 ± 0.17%, 1.88 ± 0.43%, 0.41 ± 0.08%, and 0.10 ± 0.03% of motile human sperm inside each target reservoir for flow rates of 1.5, 3, 4.5 and 6 μl/min respectively for a sample with an average density of 60 M/ml with 20 min device run time. In the process of manually counting the isolated sperm, the motile sperm entered and left the hollow regions simultaneously. Based on the obtained data, roughly 280 sperm/min entered and 80 sperm/min left; 490 sperm/min entered and 100 sperm/min left; 270 sperm/min entered and 60 sperm/min left; 130 sperm/min entered, and 50 sperm/min left each narrow curved channel at flow rates of 1.5, 3, 4.5 and 6 μl/min respectively. If we assume the number of entering sperm with a positive sign and the ones leaving it with a negative, the sum of these two values will always be positive, meaning that a higher portion of the separated sperm can use the narrow curved channels and the boundary following behavior to reach the target reservoirs. Also, another prominent factor regarding the design of this device is its capability to separate and store isolated motile sperm in the target reservoirs as long as we have flow inside the device. The designed target reservoirs can store isolated sperm without limit (see Movie S5, SI). This feature can be considered an improvement compared with previous sperm separation devices, wherein the isolated sperm’s storage space was limited, and the sperm separation process could be hampered if the mentioned space became saturated with isolated motile sperm.

Although the proposed device tried to increase the injected flow rate and the volume of the processed sample, the DNA damage of the separated sperm cells through rheotaxis and boundary-following behavior has not been investigated in this study. Therefore, suggestions for future studies using this device could be the assessment of the separated sperm cells’ DNA fragmentation index (DFI) and high DNA stainability (HDS) through the sperm chromatin structure assay (SCSA) and, as mentioned earlier, the investigation of the effects of non-Newtonian behavior of the background fluid flow and different dilution factors on separation efficiency.

## Conclusion

This paper proposes a microfluidic device for isolating motile sperm based on rheotactic and boundary-following behavior. The symmetric geometry of our device has been exploited to allow for increasing both the volume of the processed sample and injected flow rate 2 times, culminating in 4 times increase in the volume of the processed sample compared with the previous rheotaxis-based sperm separation devices. The proposed device was tested at six different flow rates of 0, 0.5, 1.5, 3, 4.5, and 6 μl/min to investigate the rheotaxis phenomenon. The results delineate that the isolated sperm are 100% motile. Also, the CASA analysis shows that the proposed device is capable of sorting different classes of sperm based on their swimming velocity. It was shown that sperm reorientation due to rheotaxis occurs if a minimum injected flow rate of 1.5 μl/min is introduced inside the device. Additionally, it was demonstrated that the inertia zone would prevail over the rheotaxis zone as the injected flow rate increases to 6 μl/min or more, which causes the motile sperm to be washed along with the strong flow at such flow rate.

## Materials and methods

### Device and sample preparation

The fresh human samples were collected from the men referred to the Gandhi IVF clinic for infertility workup. Informed consent was obtained from all participants. The ethics committee of Tarbiat Modares University (IR.MODARES.REC.1398.136) approved the study. All experiments were performed in accordance with the WHO manuals, relevant guidelines and regulations. Eight normal human sperm samples were used in this study to perform the experiments. Prior to injecting samples into the device, the microchip and microchannels were sterilized with ethanol 70%. Human tubal fluid (HTF) medium was injected into the channels for 5 min to remove alcohol from the microtubes. After filling the device with HTF medium, the device’s inlets and outlets were sealed and kept at 37 °C for 20 min so that the HTF buffer inside the device reached a steady quiescent state; then, the human samples which were diluted with the HTF buffer with the ratio of 1:2 (1 portion semen to 2 portions of HTF buffer) were injected into the device.

### Device fabrication

The microfluidic device in this study was fabricated using soft lithography in the laboratory of the MMT company (mmt-co.ir). The final design was drawn in AutoCAD software, and a photomask of the design was printed using a high-resolution chrome mask (with a precision of 10 μm). The silicon layer was cleaned and spin-coated at 300 rpm using SU8 2050 for 6 min and baked at 60 °C for 4 h. A plasma device was used to bond the microchip with glass to prevent possible leaking.

### Image and video acquisition

Images and video recordings were acquired at 30 frames per second using a Dino-Lite AM7115MZTL microscope under a magnification of 700X. Captured videos were imported in ImageJ (version 1.51j8; NIH)^45^ to be analyzed and manually track motile sperm cells. Using a MATLAB (version 2014a; MathWorks)^39^ code developed based on CASA algorithms, the motility parameters of at least 20 sperm cells that entered the narrow curved channels were evaluated for injected flow rates 1.5, 3, 4.5 and 6 μl/min. The motility parameters include VSL (velocity along the straight line between the first and last point of the path), VAP (velocity along the average path), & VCL (velocity of sperm moving along the original path). Also, PTV results were obtained by PTVLab software (version 1.2) (correlation threshold 0.8, sigma 3 pixels)^46^.

### Simulation software

A commercial CFD solver (COMSOL Multiphysics 5.4)^40^—was used to simulate the flow and particle separation in the device. The geometry of the microfluidic device was imported into the software, and then stationary laminar flow module was used to simulate flow behavior inside the device. The Navier–Stokes (Eq. (4)) and mass conservation (Eq. (5)) equations were solved with a no-slip boundary condition at the walls.

4 $$\rho \left(u.\nabla u\right)=-\nabla p+\nabla .\mu (\nabla u+{(\nabla u)}^{T})$$

5 $$\nabla .u=0,$$

where ρ is the fluid density of the modeled medium, u is the flow velocity field, p is the pressure, and μ represents the dynamic viscosity.

## Supplementary Information

Supplementary Video S1.

Supplementary Video S2.

Supplementary Video S3.

Supplementary Video S4.

Supplementary Video S5.

Supplementary Information 1.

## Data Availability

The datasets generated and analyzed during the current study are available from the corresponding author upon reasonable request.
