# Supplementary material for: A novel microfluidic device with parallel channels for sperm separation using spermatozoa intrinsic behaviors
Source: Sci Rep. 2023 Jan 21;13:1185. doi: 10.1038/s41598-023-28315-7 (PMC9867731; doi:10.1038/s41598-023-28315-7)
Supplement: Supplementary file 6 — Supplementary Information 1. [file 41598_2023_28315_MOESM6_ESM.docx]

**Supplementary information**

**A novel microfluidic device with parallel channels for sperm separation using spermatozoa intrinsic behaviors**

Ali Heydari^1^, Mohammad Zabetian Targhi^1,^ *, Iman Halvaei^2^, Reza Nosrati^3^

^1^ *Faculty of Mechanical Engineering, Tarbiat Modares University, Tehran, Iran*

^2^ *Faculty of Medical Sciences, Tarbiat Modares University, Tehran, Iran*

^3^ *Department of Mechanical and Aerospace Engineering, Monash University, Melbourne, Australia*

***^*^*** *Corresponding Author Electronic mail: zabetian@modares.ac.ir*

1. **Design principles and the microfluidic device**

The proposed device employs two main mechanisms of sperm separation inspired by what is observed inside the female reproductive tract to select competent sperm (i.e., rheotaxis and boundary-following behavior). Rheotaxis is used as the first step of the separation process. An obstacle with a diameter of 300 μm is placed inside the main channel, which has a width of 500 μm. This creates a constricted region with a width of 200 μm (Fig. S1). The reason behind placing such an obstacle is to create a low-velocity area in the obstacle's frontal area, which facilitates the exhibition of rheotactic motion by sperm cells. Moreover, the existence of a constricted region, which is another feature created by placing obstacles in the main channel, will create a barrier for strong motile sperm cells capable of swimming in the main channel to prevent them from swimming to the back of the obstacles by increasing the flow velocity to 450 μm.s^-1^. In other words, the decrease in the cross-section of the main channel will cause the flow velocity to increase and hence, ensure the entrance of the maximum possible number of motile sperm cells into the rheotaxis zone and from there into the hollow region inside the obstacles.


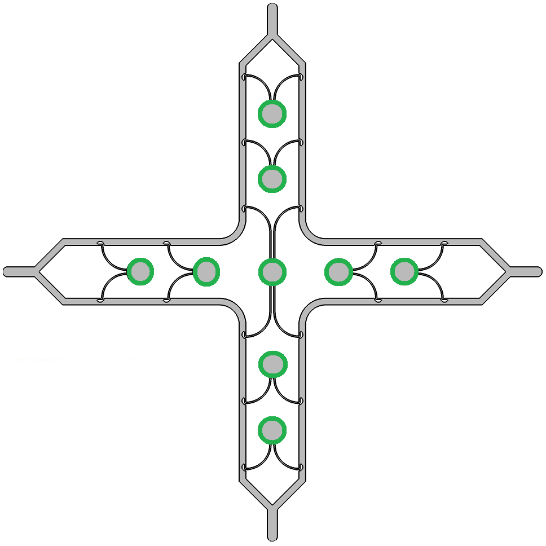

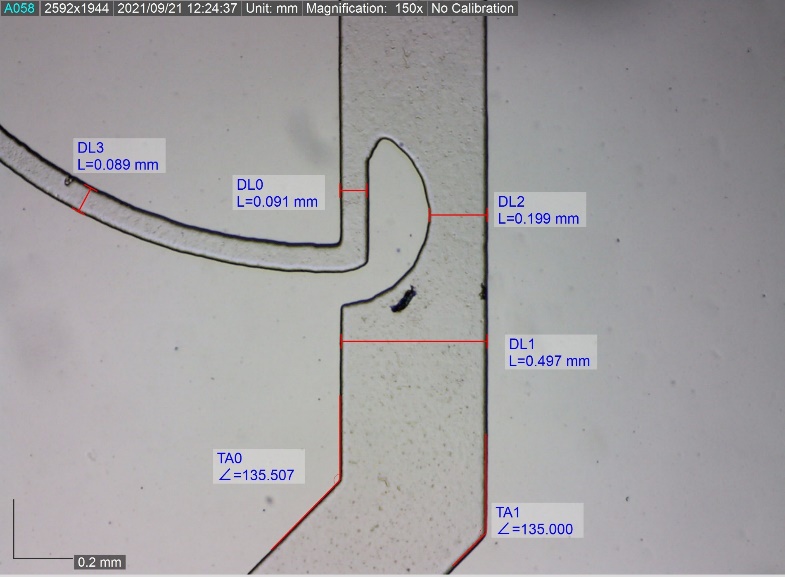


Figure S1 Detailed design of the proposed microchip. The dimensions related to each of the critical positions of the device are shown.

The boundary-following behavior is employed for the second step of the separation mechanism. Narrow curved channels with a width of 90 μm are used to connect the hollow regions inside the obstacles to the extraction reservoirs. The design of these curved channels is inspired by the curved structures inside the female reproductive organ (i.e., fallopian tubes). Although the in vivo environment of the fallopian tube is different from a simple channel with a rectangular cross-section, a curved rectangular channel would still be able to guide a larger number of sperm cells to the extraction reservoirs compared with a channel with right angles.

Furthermore, sperm cells tend to swim in random circular paths when the flow is stationary, and there is no wall in their swimming radius. In some cases, the sperm cells swimming in the proximity of a wall may become detached from it, and if the swimming space is large enough, they may even change their swimming direction [31]. Therefore, the reason behind designing the side channels narrower than the main channel is to ensure that the maximum number of sperm cells are guided toward the extraction reservoirs without changing their swimming direction in the middle.

The dimensionless number, Reynolds, should be investigated in the context of flow behavior and the interactions between particles and the flow. This number shows the dominance of inertial or viscous forces over the particles' motion. It can be expressed as equation (1), where ρ and μ are fluid density and dynamic viscosity, respectively. U_m_ is the maximum flow velocity in the channel, and D_h_ is the hydraulic diameter, obtained through equation (2), in which W is the width of the channel and H is its height. If the Re is less than 1, the viscous forces govern the particle movement. In contrast, if the Re is larger than 1, the inertial forces will dominate inside the flow and over the particles' behavior.

| (1) | $Re=\frac{\rho U_{m}D_{h}}{\mu}$ |  |
| --- | --- | --- |
| (2) | $D_{h}=\frac{2WH}{W+H}$ |  |

Two Re can be calculated for the present device; one for the plain part of the main channel and the other for the constricted region. The critical Re would then be the larger value between the two which defines the behavior of flow and particles. As shown in Table S1, the critical Re is related to the constricted region, which is equal to 0.0252 and less than 1, indicating that the flow regime is laminar and viscous forces dominate the inertial forces. The properties of background fluid are taken from Table S2.

Table S1 Detailed calculations of Re in the microfluidic device

|  | Plain part | Constricted region |
| --- | --- | --- |
| D_h_ | $\frac{2*500*100*{10}^{-12}}{600*{10}^{-6}} \tilde{=} 166.667*{10}^{-6}[m]$ | $\frac{2*200*100*{10}^{-12}}{300*{10}^{-6}} \tilde{=} 133.334*{10}^{-6}[m]$ |
| Re | $\frac{1050*125*166.667*{10}^{-12}}{2.5*{10}^{-3}}\tilde{=}0.00875$ | $\frac{1050*450*133.334*{10}^{-12}}{2.5*{10}^{-3}}\tilde{=}0.0252$ |

1. **Preliminary simulations and experiments with spherical particles**

Prior to experiments with fresh human samples, the microchip was tested with standard spherical particles to ensure the absence of any possible fabrication errors and predict the round cells' pathlines in real conditions. In order to report the results as well as analyze how the microchip works, it is necessary to observe how the particles are distributed within the microchip. One way to show how particles move inside a microchip is to investigate the particles' velocity vectors, which show the particles' velocity and moving direction. Therefore, particle velocity vectors can be carefully analyzed to obtain more insight into how particles move through the designed microchip. To perform the preliminary experiment, a suspension of poly-dispersed standard spherical particles ranging from 5 to 15 μm was used to represent the round cells in real conditions (Fig. S2A). The prepared suspension was then injected into the microchip at the flow rate of 3 μl/min to analyze the particles' velocity and motion using the particle tracking velocimetry (PTV) method.

First, the motion of particles inside the main channel and their behavior when passing over the obstacles placed inside the main channel was investigated using FEM simulation method - a commercial CFD solver (COMSOL Multiphysics 5.4) [40]. The laminar flow and particle tracing modules were used to simulate flow behavior and track particles inside the channel. The mass conservation (3) and Navier-Stokes equation (4) were solved in stationary mode, then using the data provided by the stationary solver, two types of particles with 15 (representing round cells and debris) and 5 µm (representing immotile sperm cells) in diameter were tracked inside the device (Table S2). Drag (5), Saffman lift (6), and wall-induced lift (7) forces were applied to the existing particles as the dominant forces inside the computational domain.

| (3) | $\vec{\nabla}\cdot\vec{u}=0$ |  |
| --- | --- | --- |
| (4) | $\rho\left( \vec{u}.\nabla\vec{u} \right)=-\nabla p+\nabla.\mu\left( \nabla\vec{u}+\left( \nabla\vec{u} \right)^{T} \right)$ |  |
| (5) | $\vec{F}_{D}=\frac{1}{\tau_{p}}m_{p}\left( \vec{u}-\vec{v}_{p} \right), \tau_{p}={\rho_{p}d_{p}^{2}}/{18\mu}$ |  |
| (6) | $\vec{F}_{L_{saffman}}=1.61d_{p}^{2}\sqrt{\mu\rho}\frac{(\vec{u}-\vec{v}_{p})\times\vec{\omega}_{c}}{\sqrt{\left\vert\vec{\omega}_{c} \right\vert}}, \vec{\omega}_{c}=\nabla\times\vec{u}$ |  |
| (7) | $\vec{F}_{L_{Wall-induced}}=\rho_{f}\frac{d_{p}^{4}}{16D^{2}}\beta\left( \beta G_{1}\left( s \right)+\gamma G_{2}\left( s \right) \right)\vec{n}$  $\beta=\left\vert D(\vec{n}\cdot\vec{\nabla})\vec{u}_{\vert\vert} \right\vert$  $\gamma=\left\vert D{(\vec{n}\cdot\vec{\nabla})}^{2}\vec{u}_{\vert\vert} \right\vert$  $\vec{u}_{\vert\vert}=\left( I-\left( \vec{n}\otimes\vec{n} \right) \right)\vec{u}$ |  |

Where $\vec{u}$ is fluid's velocity field, $\rho$ is density, p is pressure, $\vec{v}_{p}$ is particle's velocity, $d_{p}$ is particle's diameter, $\mu$ is fluid's dynamic viscosity, D is the distance between the parallel walls, G_1_ and G_2_ are built-in functions of the non-dimensionalized wall distance s, and n is the unit vector from the nearest point on the first parallel boundary.

Table S2 Properties of fluid and spherical particles used in simulations.

|  | Density[Kg/m^3^] | Viscosity[mPa.s] | Diameter[um] |
| --- | --- | --- | --- |
| Fluid | 1050 | 2.5 | - |
| Sperm | 1019 | - | 5 |
| Round cells | 1050 | - | 15 |

The particle tracing simulation demonstrates that 5 and 15 μm spherical particles (representing immotile sperm and round cells) cannot enter the narrow curved channels and are washed along with the flow (Fig. S2B). Also, the prepared suspension of poly-dispersed particles was injected into the channels to validate the simulation results. Figure S2C shows the pathline of an immotile particle mounted on the most critical streamline (blue line), moving extremely close to the outer periphery of an obstacle. Due to the shape of fluid flow at the entrance of the narrow curved channel, the particle is precisely placed in front of the narrow curved channel's entrance. However, since an immotile particle does not exhibit intrinsic swimming behavior, it leaves the entrance to be washed along with the fluid flow due to the drag force exerted on it (also, see movie S1).

Moreover, the motion of particles inside the device was analyzed using the PTV method to acquire more information, such as particle pathlines, velocity vectors, and statistical data for the velocity distribution of particles. Figure S3A, B show the pathlines and velocity vectors of the particles moving inside the main channel within the region of interest defined in the PTVLab software [46]. The shown pathlines and velocity vectors indicate that the particles mainly follow the flow direction and cannot enter the narrow curved channel due to the absence of innate motion. Also, statistical data on particle velocity distribution, based on the number and scattering of particle velocities in the X and Y directions, have been extracted. Based on these results, the vast majority of particles are in the velocity range of 20 to 100 μm/s, which is physically correct based on the injected flow rate into the device (i.e., 3 μl/min) (Fig. S3C, D). Altogether, the results of the preliminary experiments and simulations confirm the ability of the proposed device to separate immotile sperm cells and round cells from the initial sample by washing them along with the flow.


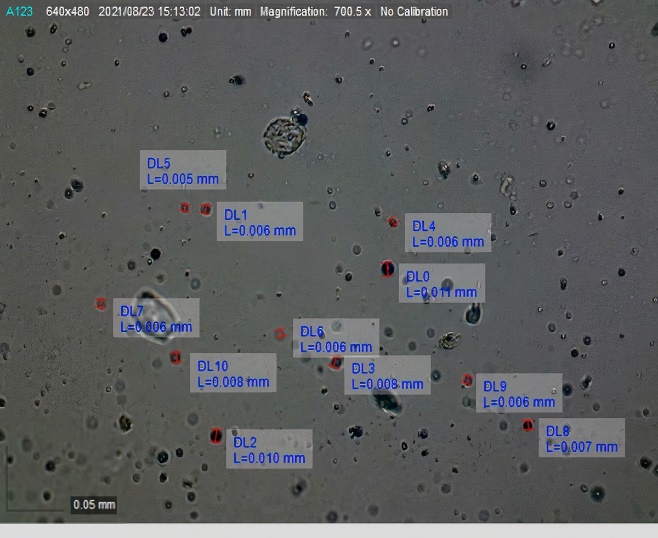

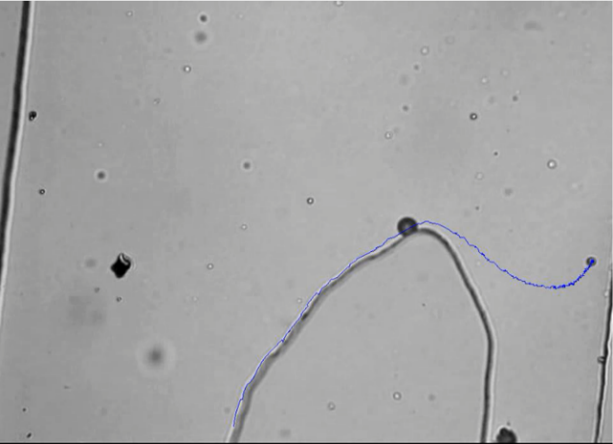


**A**

**B**

**Flow**


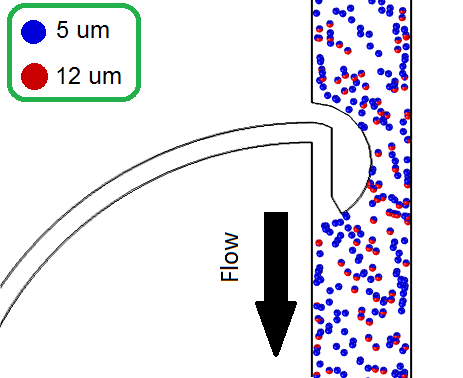


**C**

**100 μm**

Figure S2 Preliminary simulations and experiments using spherical particles. (A) The prepared suspension contains standard spherical particles ranging from 5 to 15 μm. (B) Particle tracing simulation, showing the behavior of 5 and 15 μm particles (representing immotile sperm cells and round cells, respectively) as they pass over the obstacle placed inside the main channel. (C) Manual tracking of a particle mounted on the most critical streamline moving near the obstacle's outer wall. This particle enters to some extent into the entrance of the narrow curved channel; however, since in this part, the fluid is roughly in a quiescent state, and the particle shows no intrinsic motion (such as rheotactic behavior), it cannot continue to move further into the narrow curved channel. Part B is obtained using COMSOL Multiphysics [40]. Part C is obtained using ImageJ [45].


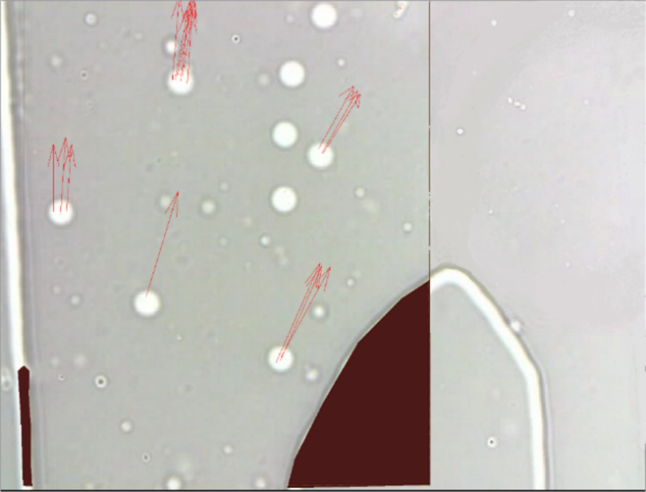

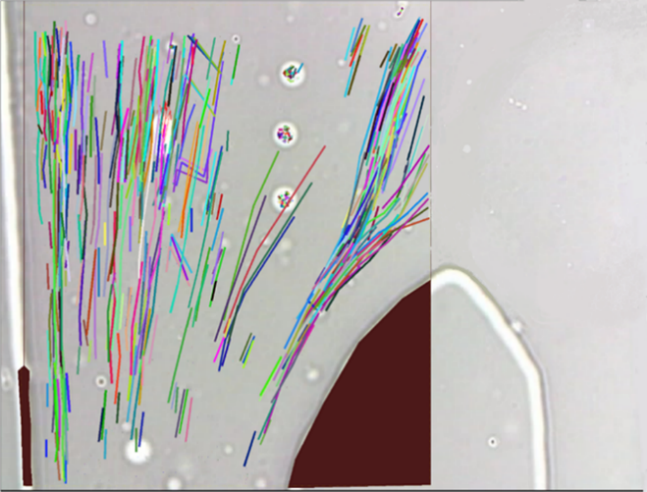


**A**

**B**

**C**

**D**

x

y

x

y

**100 μm**

**100 μm**


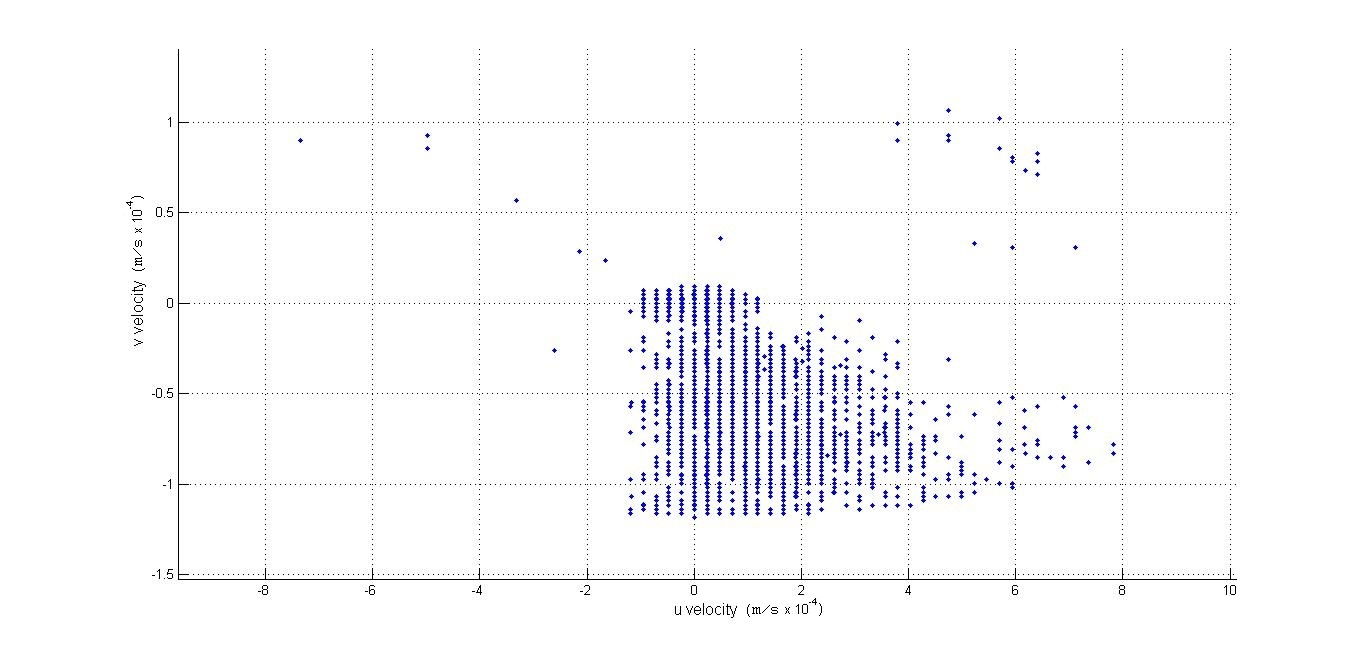

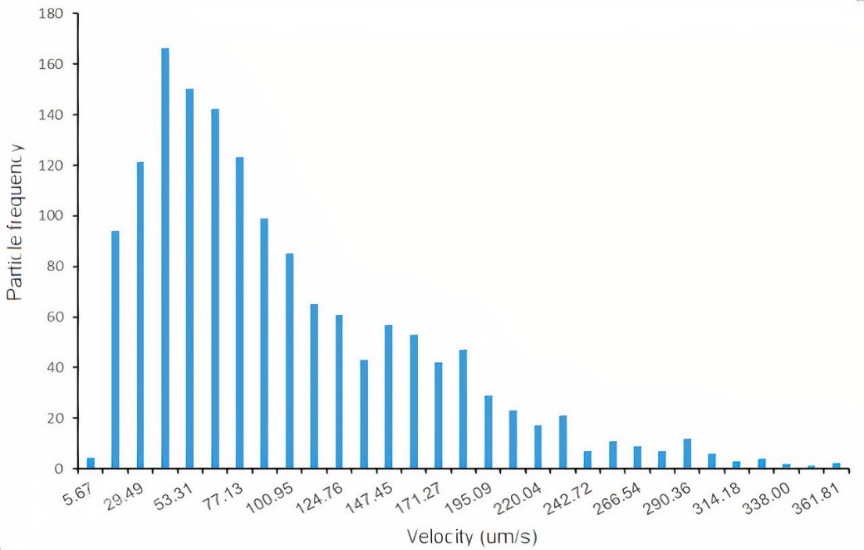


Figure S3 PTV results. (A) Pathlines of the particles existing inside the main channel within the region of interest, defined in PTVLab software. (B) Velocity vectors of the moving particles inside the main channel. (C) Scattering of the particles' velocity in X and Y directions. (D) The velocity magnitude of the investigated particles, based on their frequency, shows that the vast majority of particles are in the velocity range of 20 to 100 μm/s. Images are obtained using PTVLab [45].

Movie S1: Pathline of a particle mounted on the most critical flow streamline, moving close to the obstacle's outer wall, which cannot enter the narrow curved channel due to the absence of intrinsic motion.

Movie S2: Motile sperm cells separated through rheotaxis entering the narrow curved channel.

Movie S3: Separated motile sperm cells continue their way toward the extraction reservoir by exploiting boundary-following behavior inside the narrow curved channel (i).

Movie S4: Separated motile sperm cells continue their way toward the extraction reservoir by exploiting boundary-following behavior inside the narrow curved channel (ii).

Movie S5: Separated motile sperm cells accumulated inside the extraction reservoir.
